# Functional overload and adaptive failure during dual-tasking in Ménière’s disease

**DOI:** 10.1177/09574271261418549

**Published:** 2026-01-19

**Authors:** Mehmet Can, Soner Türüdü, Zehra Aydoğan, Ozan Bağış Özgürsoy

**Affiliations:** 1 Audiometry Program, Vocational School of Health Services, Karamanoğlu Mehmetbey University, Karaman, Türkiye; 2 Audiology and Speech Disorders PhD Program, Institute of Health Sciences, Ankara University, Ankara, Türkiye; 3 Department of Otorhinolaryngology/Head and Neck Surgery, University Medical Center Groningen, 10173 University of Groningen, Groningen, Netherlands; 4 Research School of Behavioral and Cognitive Neuroscience, University of Groningen, Groningen, Netherlands; 5 Department of Audiology, Faculty of Health Sciences, Ankara University, Ankara, Türkiye; 6 Department of Otorhinolaryngology, Faculty of Medicine, Ankara University, Ankara, Türkiye

**Keywords:** Ménière’s disease, vestibular disorder, dual-tasking, cognitive-motor interference, postural control

## Abstract

**Background:**

Ménière’s disease (MD) impairs cognitive function, yet the dynamics of cognitive-motor interference are not well understood. This study investigated dual-task performance in MD, hypothesizing a greater performance cost for patients compared to healthy controls under combined cognitive and postural loads.

**Method:**

Twenty-nine patients with MD and 29 healthy controls participated in a dual-task paradigm that combined computerized dynamic posturography (CDP) with a cognitive battery assessing visuospatial, executive, and working memory functions. Performance was measured across Sensory Organization Test (SOT) conditions of increasing difficulty and analyzed using mixed-effects models.

**Results:**

Significant dual-task interference was found in the MD group only for reaction times on two cognitive tasks (Mental Rotation and Shifting Attention), where increasing postural difficulty disproportionately slowed performance relative to controls. No dual-task effects were found in the other four cognitive tasks (Visual Stroop Task, Backward Digit Span Task, Corsi Block Task, and Symbol Digit Coding Task). Importantly, concurrent cognitive tasks did not significantly impair postural stability in either group. Patients also exhibited baseline deficits specifically on visuospatial working memory tasks.

**Conclusion:**

Individuals with MD demonstrate selective cognitive-motor interference in demanding visuospatial and executive tasks, without concomitant deterioration in postural control under dual-task conditions. This suggests a posture first strategy and highlights a quantifiable, task-specific cognitive burden that is a clinically relevant feature of the disorder. Dual-task paradigms provide a sensitive method for assessing this impact, with clear implications for comprehensive patient evaluation and management.

## Introduction

Ménière’s disease (MD) is a multifaceted disorder influenced by genetic and environmental factors. Histopathological studies have linked MD to endolymphatic hydrops (EH), characterized by the accumulation of endolymph within the cochlear duct and vestibular organs.^1^ The finely tuned sensory perception system can be adversely affected by EH, often leading to unpredictable episodes of vertigo. EH may also affect the auditory system, resulting in permanent sensorineural hearing loss (SNHL), tinnitus (or buzzing), and a feeling of pressure or fullness in the ear.^2^

Patients with various vestibular disorders (VDs) experience vertigo, postural control problems, and nausea. Among these conditions, MD provides a unique framework for studying cognitive-motor interference. The erratic, intermittent nature of its symptoms may necessitate a continuous, cognitively taxing process of sensory re-weighting and adaptation, even during asymptomatic phases. Consistent with this, cognitive impairments have been widely observed in individuals with VD, including deficits in attention, processing speed, executive function, working memory, and visuospatial memory.^3–6^ Therefore, the fluctuating nature of MD provides an opportunity to study not only the cost of vestibular loss but also the continuous cognitive burden of sensory unpredictability.

One explanation for impaired visuospatial memory in MD involves hippocampal atrophy.^7^ The hippocampus plays a crucial role in storing and encoding spatial information, thereby contributing to spatial memory and navigation.^8^ In addition, cognition is supported by a broad network of vestibular-cortical pathways, including critical areas in the parietal and temporal cortices, the cingulate gyrus, and the cerebellum.^9^ Disrupted sensory input from these pathways in VD may be associated with cognitive impairment.

Maintaining postural control requires the use of attentional resources, thus involving higher levels of cognitive processing.^10^ Patients with VD may have less cognitive reserve available for other tasks due to the increased effort required to maintain motor skills and postural stability. Kahneman’s model suggests that higher effort may reduce attentional resources available for concurrent cognitive tasks.^11^ Consequently, conditions that require more complex postural control may demand greater attentional capacity, potentially leading to a cognitive-motor interaction.^12^

Investigations of the cognitive-motor interplay using dual-task paradigms have yielded varied results. In clinical populations, a common finding is a cost to postural control, even when performance on a concurrent cognitive task is preserved.^13^ However, the vulnerability of cognitive performance appears task-dependent, with significant impairments observed in demanding executive and visuospatial tasks but not necessarily in others.^3^ This complexity is mirrored in studies of healthy individuals, where the effect of increasing postural challenge on cognition is inconsistent. While some studies report no change in cognitive performance,^13^ other research shows a distinct decline in spatial processing as balance demands rise.^14^ Given these inconsistencies across both healthy and patient populations, the specific patterns of interference in MD warrant closer investigation, particularly for high-level cognitive domains such as visuospatial processing and executive control.

Therefore, the aim of the current study was to investigate dual-task effects in patients with MD in comparison to healthy controls. We hypothesized that the MD group would exhibit not just a significantly greater dual-task cost, but a particular pattern of adaptive failure. Specifically, we predicted that as postural difficulty increased, MD patients would be unable to implement the efficient processing strategies seen in healthy controls, which leads to marked performance decline in tasks demanding visuospatial abilities and executive functions. Such a finding would suggest a functional overload on the specific neural networks, including vestibulo-hippocampal and parieto-cortical pathways, that are presumed to be compromised in MD and are essential for both spatial cognition and postural control.

## Method

### Participants

Permission to conduct this study was obtained from the Ankara University Faculty of Medicine Ethics Committee (Code: I02-144-24), and all participants provided written informed consent. The sample size was determined by a priori power analysis, which established that our final sample of 29 participants per group was adequately powered to detect a medium-sized interaction effect with 90% power.

A total of 29 patients with a definitive diagnosis of MD, according to the Bárány Society criteria,^15^ were recruited during an asymptomatic period. The age range for all participants was 18 to 60 years. Exclusion criteria for the MD group were comorbid eye disease, oscillopsia, suspected central pathology, or limb loss. Exclusion criteria for the control group were any known otological, ophthalmological, or neurological disorders. Participants scoring below 25 points on the Turkish-validated Standardized Mini-Mental Test (SMMT) were excluded from both groups.^16^

### Procedure and materials

Participants attended a single experimental session. Upon arrival, they underwent preliminary assessments, including a detailed medical history, audiological evaluation, and baseline questionnaires, before proceeding to the main experimental protocol. The audiological evaluation consisted of pure-tone audiometry (125-8000 Hz) and immittance testing to exclude individuals with external or middle ear pathology. Before the balance assessment, the Cognitive Failure Questionnaire (CFQ) was administered to all participants. The CFQ, initially developed by Broadbent et al. (1982), assesses everyday cognitive lapses related to perception, memory, and attention.^17^ Its four-point Likert version was validated in Turkish in 2020.^18^ Responses are scored as never = 0, rarely = 1, occasionally = 2, and often = 3 points, with a total possible score of 75. Higher CFQ scores indicate more pronounced cognitive impairment.

### Computerized Dynamic Posturography and dual-task protocol

To investigate cognitive-motor interactions, participants underwent evaluations using the NeuroCom^®^ Balance Manager Computerized Dynamic Posturography (CDP) device (Natus Medical Inc., Seattle, WA, USA).^19^ Sensory Organization Tests (SOTs) were conducted to measure balance under both single-task (balance only) and dual-task (balance plus a concurrent cognitive task) conditions. In all dual-task conditions, participants were explicitly instructed to prioritize maintaining their balance over performance on the cognitive task.

### Sensory Organization Test

The SOT is considered the gold standard for evaluating the integration of the three sensory systems contributing to postural control. It assesses balance under six various conditions (C1-C6) involving combinations of eyes open or eyes closed, fixed ground or sway-referenced ground, and fixed vision or sway-referenced vision.^20^ Each condition is repeated three times for 20 s. The device calculates an Equilibrium Score (ES) for each trial based on the peak-to-peak anteroposterior (AP) sway angle relative to the theoretical limit of stability (12.5°). The ES is calculated using the following formula:

$$ES=\frac{12.5{}^{\circ}-\left(\theta \;\max-\theta \;\min\right)}{12.5{}^{\circ}}\;x\;100$$

where θ_max and_ θ_min_ represent the maximum and minimum sway angles during the 20-second trial. A score of 100 indicates perfect stability (no sway), while 0 indicates a fall or sway approaching the limit of stability.

In addition to the ES for each condition, a Composite Score (weighted average of all conditions) and specific sensory analysis ratios were calculated to quantify the reliance on specific sensory systems, including somatosensory (SOM = C2/C1), visual (VIS = C4/C1), vestibular (VEST = C5/C1) and visual preference (PREF = (C3+C6)/(C2+C5)).

In the single-task condition, the SOT was performed without any concurrent cognitive task. For the dual-task protocol, participants performed six different cognitive tasks, including the Visual Stroop Task (VST), Backward Digit Span Task (BDST), Corsi Block Task (CBT), Mental Rotation Task (MRT), Symbol Digit Coding Task (SDCT), and Shifting Attention Task (SAT), presented on the CPD screen (Figure 1) during the eye-open SOT conditions (C1, C3, C4, C6). In certain tasks, participants used a handheld controller with left and right buttons for responses. The SOT was repeated six times, each time paired with one of these tasks.

**Figure 1.**
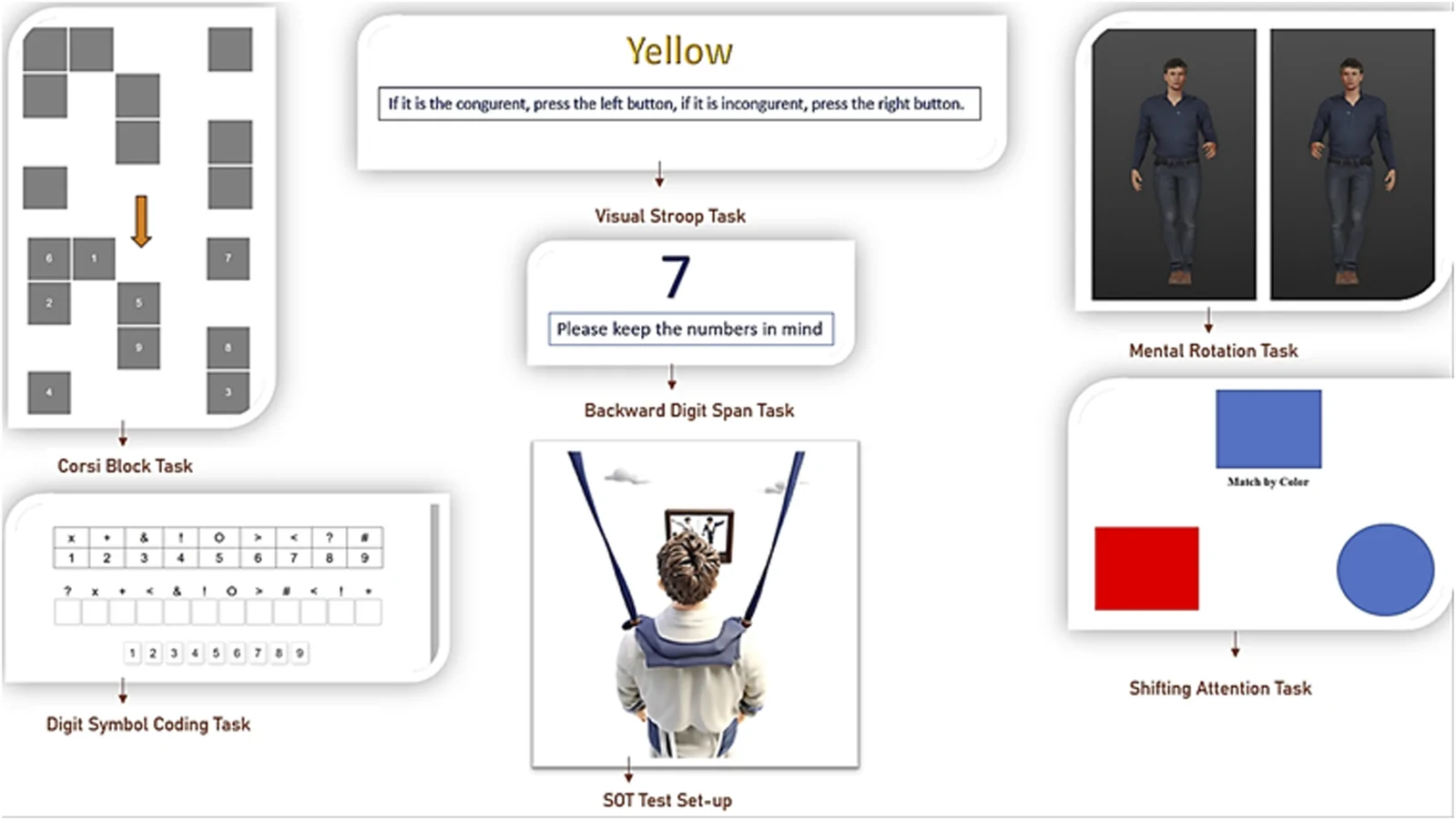
Example SOT Setup and Cognitive Tasks. This figure illustrates how participants stand in a safety harness while performing the SOT in both single- and dual-task conditions. The center image shows the SOT platform, and the surrounding panels depict screenshots of the six cognitive tasks integrated into the dual-task protocol: The CBT (top left), VST (top center), MRT (top right), SAT (right center), SDCT (bottom left), and BDST (center). During each dual-task trial, participants respond to the on-screen tasks while the SOT measures balance performance.

During eye-open conditions (C1, C3, C4, and C6), participants carried out the visual-cognitive tasks described below. During eye-closed conditions (C2 and C5), they performed backward-counting tasks (counting backward from 200 by 3 s in C2 and by 4 s in C5). The purpose of these non-visual tasks was to ensure consistent cognitive engagement throughout all SOT conditions, thereby minimizing potential confounding effects of mental rest or fatigue between trials. As this study’s primary focus is on visual-cognitive interference, data from these counting tasks were not analyzed. The order of the task conditions was randomized to minimize learning effects. Participants were given a 5- to 10-minute break after every two tasks to reduce fatigue.

### Cognitive tasks

All cognitive tasks were programmed in JsPsych 7.3^21^ and stored on a JATOS server.^22^ To ensure compatibility with the 20-second trial duration of the SOT, the tasks were adapted from original sources^5,12,23^ primarily by reducing the number of stimuli presented per trial while preserving the core cognitive demands of each test. The feasibility of these adapted tasks and the absence of any floor or ceiling effects were confirmed in a pilot study conducted with a small sample of healthy adults (n = 5) before the main experiment. Upon finishing each task, the participant received on-screen feedback indicating completion.

The Stroop task assesses complex attention, executive functions, and cognitive flexibility.^24^ Four color words (blue, red, green, and yellow) appeared in matching (congruent) or mismatching (incongruent) ink colors. Participants pressed the left button for congruent pairs and the right button for incongruent pairs. Sixteen stimuli (eight congruent, eight incongruent) were presented. Although the interstimulus interval was set at 200 ms, the next stimulus appeared immediately after each response. At the end of the task, the percentage of correct responses and reaction times was recorded.

The Digit Span task evaluates attention capacity, short-term memory, and working memory.^25^ In this study, the backward digit span version was administered. Participants were presented with digit sequences on a screen at 900-ms intervals, starting with two digits, then three, and then four. After each sequence, they were instructed, “Now repeat the numbers in reverse.” Points were awarded for each correctly reversed sequence (2 points for two digits, 3 points for three digits, and 4 points for four digits).

The CBT assesses visuospatial working memory.^26^ To avoid introducing an extra motor component, participants were shown nine squares containing hidden numbers. First, three squares in different positions flashed for 500 ms each. Participants then recalled and stated the numbers in the order they had flashed. The sequence verbally stated by the participant was recorded by the researcher, and scoring was based on this record. The same procedure was repeated with four and then five squares. Two trials were performed for each sequence length. Points were awarded based on the total number of squares correctly recalled (3 points for three squares, 4 points for four squares, and so on).

The MRT demonstrates how mental imagery is used in thinking and assesses visuospatial skills.^12^ Pairs of 3D human figures were displayed at angles of 0°, 60°, 120°, or 180°, with varying arm or leg positions. Participants clicked the left button if the two figures were mirror images and the right button if they were not. Twenty stimuli were presented (10 mirror-image pairs, 10 non-mirror pairs). The next stimulus appeared immediately after each response. Each correct answer was scored as 1 point, and reaction times were recorded.

The SDCT measures processing speed and oculomotor scanning.^27^ Nine digits paired with different geometric figures were shown at the top of the screen, and twelve items containing these symbols appeared at the bottom. Participants either checked the top reference or relied on memory to match each symbol with its corresponding digit. One point was awarded for each correct match.

Based on the Wisconsin Card Sorting Test, the SAT assesses frontal lobe functions, including rule learning and concept formation.^28^ One shape (square or circle, in blue or red) appeared at the top of the screen, and two shapes were shown at the bottom. The instruction was either “match by color” or “match by shape.” Fourteen stimuli were presented (seven matching by color and seven matching by shape), each displayed for 1750 ms. Participants pressed the left or right arrow key to select the correct match. The percentage of correct responses and reaction times were recorded at the end of the task.

### Statistical analysis

All analyses were performed using R Studio,^29^ with a significance level (α) of 0.05. Baseline demographic and clinical characteristics were compared between groups using independent samples t-tests or the Mann-Whitney U test, as dictated by the data’s normality, assessed with the Shapiro-Wilk test. For categorical data such as the distribution of education levels, Pearson’s chi-square test was employed. To analyze the effects of dual-tasking on cognitive and balance performance, a series of mixed-effects models was employed. The distribution of the dependent variable determined the choice of model. Specifically, Linear Mixed-Effects Models (LMMs) were used for continuous outcomes with normally distributed residuals, such as reaction times, balance scores, and point-based cognitive scores. For accuracy scores, which represent binomial data (i.e., the number of correct responses out of a fixed number of trials), Generalized Linear Mixed-Effects Models (GLMM) with a binomial distribution were utilized. For all models, Group (MD vs control), Condition (the specific dual-task balance challenge), and the Group-Condition interaction were included as fixed effects. Participants’ age and ordinal education score were also included as covariates in all models to control for the potential confounding influence of educational background and aging. To account for the non-independence of repeated measures from the same individual, a random intercept for each participant was included in all models. Our primary hypothesis regarding differential dual-task costs was tested by examining the significance of the interaction term between Group and Condition. Significant interactions were explored using post hoc comparisons with the Bonferroni correction. Finally, Spearman’s correlation coefficient was used to assess relationships between clinical variables and performance scores, as well as to explore the potential confounding influence of education level on performance outcomes.

## Results

### Participant characteristics

A total of 29 patients with MD (20 female, nine male; mean age: 46.60 ± 13.0 years) and 29 healthy controls (19 female, 10 male; mean age: 41.53 ± 13.0 years) participated in this study. There were no significant between-group differences in age [*t*(56) = −1.44, *p* = 0.16] or sex distribution (*p* = 1.00). The overall distribution of education levels, categorized according to the Turkish education system, also did not differ between the groups (Χ^2^(6) = 11.2, p = 0.08). The control group consisted of participants with middle school (n = 2), high school (n = 4), bachelor’s (n = 12), associate’s (n = 3), and master’s (n = 8) degrees. The MD group consisted of participants with primary school (n = 4), high school (n = 5), bachelor’s (n = 15), associate’s (n = 2), master’s (n = 2), and doctoral (n = 1) degrees. For clarity, a high school degree in this context corresponds to 12 years of formal education and a bachelor’s degree to 16 years.

The mean hearing thresholds for both groups are presented in Figure 2. As expected, patients with MD exhibited significantly greater hearing loss than controls, with a median PTA of 35.0 [20.0–58.0] dB HL in their most affected ear, compared to 5.0 [3.3–10.8] dB HL in controls (*W* = 29.5, *p* < 0.001). Based on the standards proposed by Clark (1981), hearing levels in the MD group were classified as normal in 5 patients (SNHL at low frequencies), slight loss in 7, and mild-to-severe in the remaining 17. All participants scored within the normal range on the SMMT, and there was no significant difference between the MD and control groups on this measure (*W* = 406, *p* = 0.81). Similarly, self-reported cognitive complaints, as measured by the CFQ, did not differ significantly (*W* = 373.5, *p* = 0.47). The demographic and baseline characteristics of the participants are summarized in Table 1.

**Figure 2.**
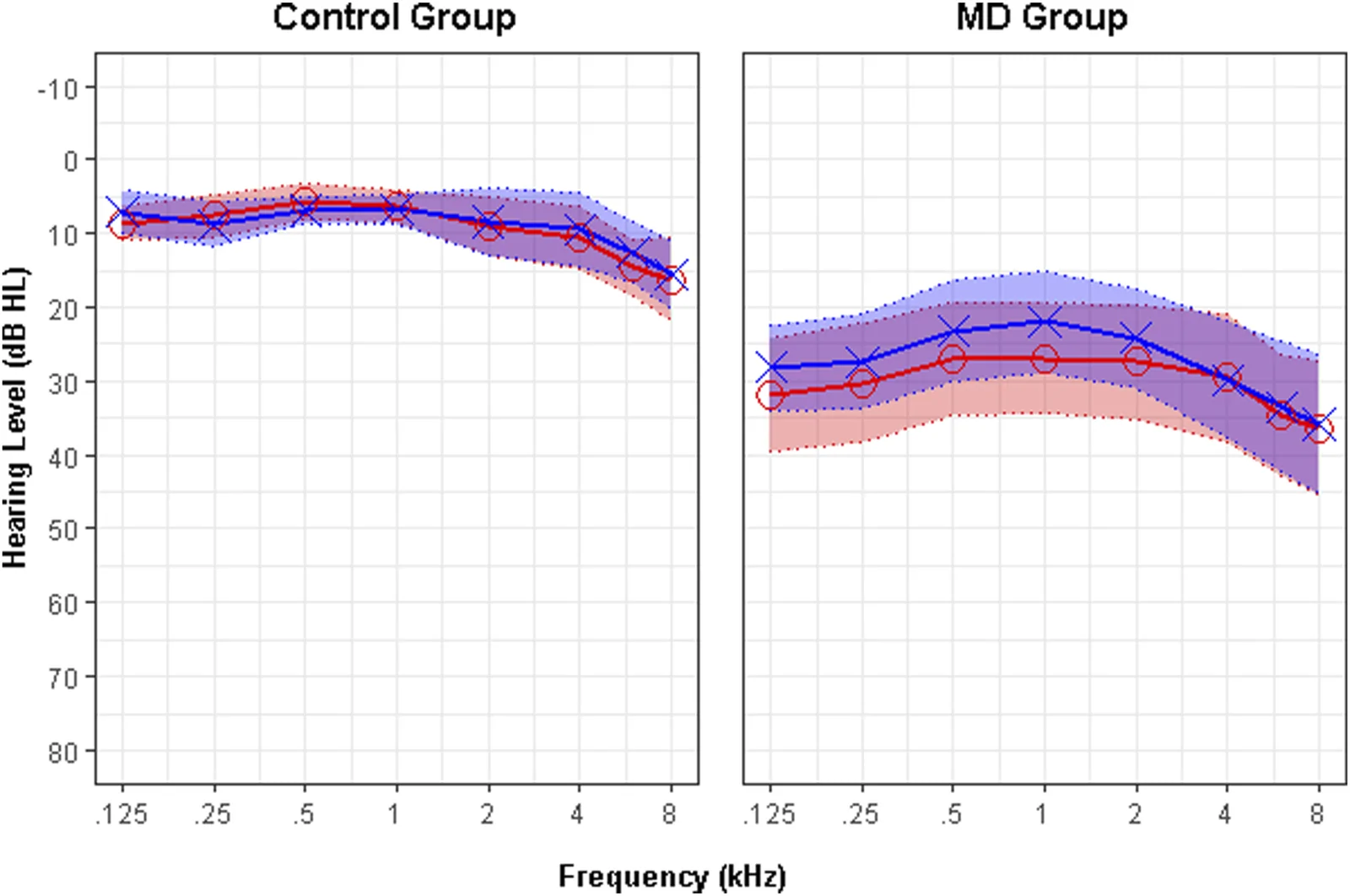
Mean audiometric thresholds for the control and MD groups. The graphs display average hearing thresholds for the right ear (red circles) and the left ear (blue crosses). Note that for the clinical analyses presented in the text, data from each MD patient’s more-affected ear was used, irrespective of the side shown here. The shaded areas represent 95% confidence intervals.

**Table 1.** Participant demographics and baseline comparisons.

Characteristic	Control group (n = 29)	MD group (n = 29)	Statistic
Age (years), mean ± SD	41.53 ± 13.0	46.60 ± 13.0	*t*(56) = −1.44, *p* = 0.16
Sex (female/male), n (%)	19 (65.5%)/10 (34.5%)	20 (69.0%)/9 (31.0%)	*p* = 1.00^a^
SMMT score, median [IQR]	30 [27–30]	30 [28–30]	*W* = 406, *p* = 0.81
CFQ score, median [IQR]	26 [22.0–38.0]	29.0 [21.0–39.0]	*W* = 373.5, *p* = 0.47
PTA (dB HL), median [IQR]	5.0 [3.3–10.8]	35.0 [20.0–58.0]	*W* = 29.5, *p* < 0.001

*Note.* SD = standard deviation; IQR = interquartile range; SMMT = Standardized Mini-Mental Test; CFQ = Cognitive Failure Questionnaire; MD = Meniere’s disease; PTA = Pure-Tone Average (0.5, 1, and 2 kHz).

^a^Fisher’s exact test was used.

For the MD group, the mean disease duration was 6.96 ± 4.9 years. Clinical assessment revealed that 16 patients had unilateral (8 right-sided and 8 left-sided) and 13 had bilateral MD.

## Dual-task performance

### Cognitive performance

Descriptive statistics for all cognitive performance measures are provided in Table 2. To formally test the dual-task effects, a series of mixed-effects models was conducted, which included age and education level as covariates to control for their potential influence on the results. GLMMs were employed to analyze accuracy data, while LMMs were utilized for continuous outcomes, including reaction times and point-based scores. The comprehensive results of these analyses are presented in Table 3.

**Table 2.** Descriptive statistics for cognitive performance.

Task	Measure	Control group (n = 29)				MD group (n = 29)			
		C1 (x ± SD)	C3 (x ± SD)	C4 (x ± SD)	C6 (x ± SD)	C1 (x ± SD)	C3 (x ± SD)	C4 (x ± SD)	C6 (x ± SD)
VST	Reaction time (ms)	997.75 ± 141.26	1007.31 ± 203.82	981.78 ± 212.97	973.83 ± 194.24	1077.94 ± 184.40	1040.73 ± 186.29	1029.32 ± 159.43	1015.83 ± 171.68
	Accuracy (%)	89.34 ± 10.72	90.65 ± 11.86	89.11 ± 11.63	92.40 ± 9.40	78.97 ± 23.20	87.71 ± 19.43	87.05 ± 20.32	88.47 ± 18.99
BDST	Score (points)	15.03 ± 2.64	15.17 ± 3.30	14.48 ± 3.67	13.41 ± 2.37	12.93 ± 3.00	13.62 ± 2.72	13.45 ± 3.27	12.34 ± 2.92
CBT	Score (points)	19.62 ± 4.25	18.41 ± 4.02	18.41 ± 4.00	16.62 ± 4.44	15.90 ± 4.31	13.83 ± 4.05	15.76 ± 4.02	13.03 ± 2.78
MRT	Reaction time (ms)	1892.10 ± 527.24	1840.52 ± 633.91	1686.59 ± 434.17	1593.51 ± 405.94	1866.59 ± 478.70	1864.49 ± 417.09	1882.28 ± 456.07	1929.90 ± 525.86
	Accuracy (points)	5.19 ± 2.44	5.50 ± 2.52	5.31 ± 3.32	6.05 ± 3.10	3.72 ± 1.93	3.76 ± 2.20	3.69 ± 2.00	3.45 ± 1.90
SDCT	Score (points)	9.93 ± 2.33	10.38 ± 1.74	10.52 ± 2.18	9.90 ± 2.57	9.31 ± 2.69	8.83 ± 3.11	9.45 ± 2.53	8.62 ± 2.87
SAT	Reaction time (ms)	1001.60 ± 216.17	992.16 ± 170.63	1040.64 ± 178.70	970.98 ± 275.95	956.48 ± 242.30	932.00 ± 236.88	900.62 ± 264.65	984.41 ± 181.81
	Accuracy (%)	82.57 ± 20.94	82.77 ± 18.59	87.76 ± 18.11	87.15 ± 16.04	70.76 ± 23.37	70.22 ± 27.14	75.35 ± 25.23	74.62 ± 17.91

*Note. Values are presented as Mean ± SD. C1, C3, C4, and C6 refer to SOT conditions with increasing postural difficulty.* X; Mean, VST = Visual Stroop Task; BDST = Backward Digit Span Task; CBT = Corsi Block Task; MRT = Mental Rotation Task; SDCT = Symbol Digit Coding Task; SAT = Shifting Attention Task.

**Table 3.** Summary of mixed-effects models for cognitive performance.

Task	Dependent variable	Main effect of group	Main effect of condition	Interaction (group-condition)	Main effect of education	Main effect of age
VST	Accuracy (GLMM)	Χ^2^(1) = 1.78, *p* = 0.182	Χ^2^(3) = 5.35, *p* = 0.148	Χ^2^(3) = 6.61, *p* = 0.085	Χ^2^(1) = 6.70, *p* = 0.010*	Χ^2^(1) = 21.05, *p* < 0.001**
	Reaction time (LMM)	*F*(1, 54) = 0.02, *p* = 0.899, $\eta_{p}^{2}$ < 0.01	*F*(3, 168) = 1.76, *p* = 0.158, $\eta_{p}^{2}$ = 0.03	*F*(3, 168) = 0.50, *p* = 0.682, $\eta_{p}^{2}$ < 0.01	*F*(1, 54) = 0.31, *p* = 0.578, $\eta_{p}^{2}$ < 0.01	*F*(1, 54) = 67.35, *p* < 0.001**, $\eta_{p}^{2}$ = 0.55
BDST	Score (LMM)	*F*(1, 54) = 2.92, *p* = 0.093, $\eta_{p}^{2}$ = 0.05	*F*(3, 168) = 4.83, *p* = 0.003**, $\eta_{p}^{2}$ = 0.08	*F*(3, 168) = 0.72, *p* = 0.541, $\eta_{p}^{2}$ = 0.01	*F*(1, 54) = 2.70, *p* = 0.106, $\eta_{p}^{2}$ = 0.05	*F*(1, 54) = 8.22, *p* = 0.006**, $\eta_{p}^{2}$ = 0.13
CBT	Score (LMM)	*F*(1, 54) = 18.36, *p* < 0.001**, $\eta_{p}^{2}$ = 0.25	*F*(3, 168) = 9.58, *p* < 0.001**, $\eta_{p}^{2}$ = 0.15	*F*(3, 168) = 0.92, *p* = 0.430, $\eta_{p}^{2}$ = 0.02	*F*(1, 54) = 8.19, *p* = 0.006**, $\eta_{p}^{2}$ = 0.13	*F*(1, 54) = 0.00, *p* = 0.947, $\eta_{p}^{2}$ < 0.01
MRT	Accuracy (GLMM)	Χ^2^(1) = 0.10, *p* = 0.748	Χ^2^(3) = 1.05, *p* = 0.789	Χ^2^(3) = 1.12, *p* = 0.772	Χ^2^(1) = 2.64, *p* = 0.104	Χ^2^(1) = 0.94, *p* = 0.332
	Reaction time (LMM)	*F*(1, 54) = 0.81, *p* = 0.373, $\eta_{p}^{2}$ = 0.01	*F*(3, 168) = 1.47, *p* = 0.224, $\eta_{p}^{2}$ = 0.03	*F*(3, 168) = 3.28, *p* = 0.022*, $\eta_{p}^{2}$ = 0.06	*F*(1, 54) = 1.22, *p* = 0.275, $\eta_{p}^{2}$ = 0.02	*F*(1, 54) = 0.82, *p* = 0.368, $\eta_{p}^{2}$ = 0.01
SDCT	Score (LMM)	*F*(1, 54) = 1.11, *p* = 0.297, $\eta_{p}^{2}$ = 0.02	*F*(3, 168) = 2.49, *p* = 0.062, $\eta_{p}^{2}$ = 0.04	*F*(3, 168) = 1.09, *p* = 0.353, $\eta_{p}^{2}$ = 0.02	*F*(1, 54) = 4.36, *p* = 0.041*, $\eta_{p}^{2}$ = 0.07	*F*(1, 54) = 29.32, *p* < 0.001**, $\eta_{p}^{2}$ < 0.35
SAT	Accuracy (GLMM)	Χ^2^(1) = 2.40, *p* = 0.121	Χ^2^(3) = 8.34, *p* = 0.040*	Χ^2^(3) = 0.69, *p* = 0.875	Χ^2^(1) = 16.74, *p* < 0.001**	Χ^2^(1) = 9.70, *p* = 0.002**
	Reaction time (LMM)	*F*(1, 54) = 0.27, *p* = 0.608, $\eta_{p}^{2}$ < 0.01	*F*(3, 168) = 0.17, *p* = 0.919, $\eta_{p}^{2}$ < 0.01	*F*(3, 168) = 2.74, *p* = 0.045, $\eta_{p}^{2}$ = 0.05	*F*(1, 54) = 11.72, *p* < 0.001**, $\eta_{p}^{2}$ = 0.18	*F*(1, 54) = 0.41, *p* = 0.524, $\eta_{p}^{2}$ < 0.01

*Note.* VST = Visual Stroop Task; BDST = Backward Digit Span Task; CBT = Corsi Block Task; MRT = Mental Rotation Task; SDCT = Symbol Digit Coding Task; SAT = Shifting Attention Task. GLMM = Generalized Linear Mixed-Effects Model; LMM = Linear Mixed-Effects Model. Statistics are reported as Χ^2^(df) for GLMM and *F*(df, df) for LMM. *p < 0.05; **p < 0.01.

The mixed-effects models confirmed our primary hypothesis regarding cognitive-motor interference. A significant Group by Condition interaction was found for reaction time on the MRT [*F*(3, 168) = 3.28, *p* = 0.022, $\eta_{p}^{2}$ = 0.06], and the SAT [*F*(3, 168) = 2.74, *p* = 0.045, $\eta_{p}^{2}$ = 0.05].

To dissect the significant interaction on the MRT, post hoc comparisons were performed using the Bonferroni correction. The results indicated that the performance of the two groups varied as postural difficulty escalated. No significant difference in reaction time was observed between the groups under easier conditions (C1, C3, and C4; all *p* > 0.05). However, the control group demonstrated a significantly faster reaction time compared to the MD group in the most challenging balance condition (C6; *p* = 0.018). Within-group analyses further clarified that the control group’s performance significantly improved between conditions C1 and C6 (*p* = 0.008). In contrast, the MD group’s reaction time showed no significant change across any of the conditions (*p* = 1.00). For the significant interaction on the SAT, subsequent post hoc tests did not reveal any pairwise differences (all *p* > 0.05). This suggests that the interaction on the SAT is driven by a more subtle and complex pattern of performance across conditions, as seen in Figure 3 (left panel), rather than a single specific difference.

**Figure 3.**
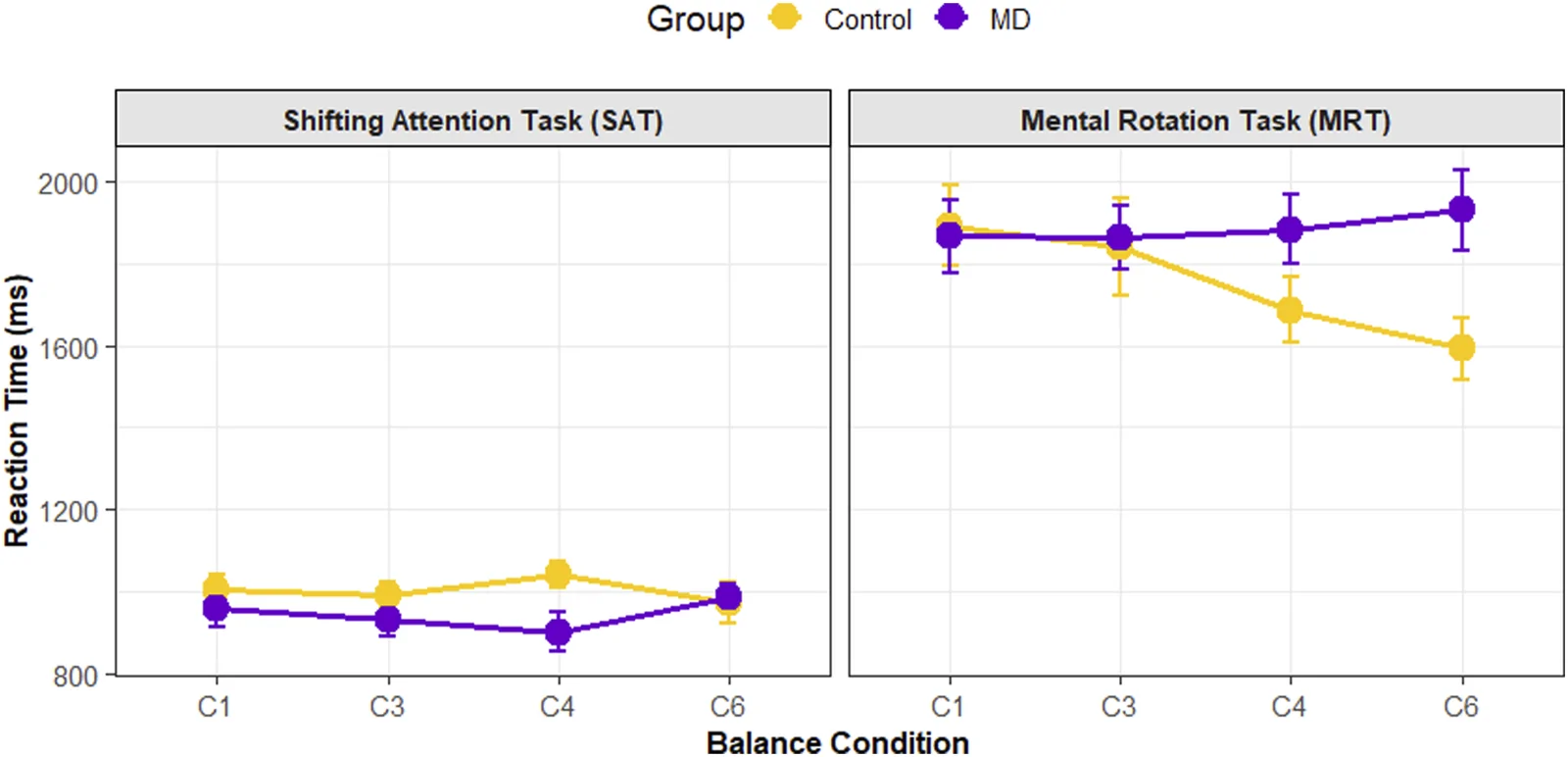
Reaction times (ms) for the Control (yellow) and MD (purple) groups during the SAT (left panel) and the MRT (right panel) across four balance conditions of increasing difficulty (C1-C6). Error bars represent the standard error of the mean.

Furthermore, significant main effects of Group were observed. After accounting for age and education, the MD group performed significantly worse than controls on the CBT [*F*(1, 54) = 18.36, *p* < 0.001, $\eta_{p}^{2}$ = 0.25]. While the MD group scored lower on the BDST, the difference was not significant [*F*(1, 54) = 2.92, *p* = 0.093, $\eta_{p}^{2}$ = 0.05].

Education level and age were significant predictors of performance across most cognitive tasks. While age showed a strong effect, particularly on reaction time measures (e.g., VST and SDCT) and accuracy on VST and SAT, education remained a significant predictor primarily for accuracy and working memory scores (e.g., CBT and SAT). A main effect of Condition was also observed for several measures, including scores on the BDST and CBT, as well as accuracy on the SAT (see Table 3 for all statistics).

### Balance performance

Descriptive statistics for balance scores under both single-task and dual-task conditions are presented in Table 4. Both groups demonstrated a decline in balance performance as postural challenges increased (e.g., from C1 to C6). The effects of dual-tasking and increasing postural difficulty on balance performance were formally tested with an LMM.

**Table 4.** Balance performance during dual-task conditions.

Cognitive task	Group	C1 (x ± SD)	C3 (x ± SD)	C4 (x ± SD)	C6 (x ± SD)
Single task	Control	94.86 ± 2.03	93.41 ± 2.84	89.55 ± 6.22	78.50 ± 9.70
	MD	94.79 ± 2.03	92.18 ± 4.51	86.70 ± 10.90	71.24 ± 15.14
VST	Control	95.33 ± 1.60	93.24 ± 3.57	90.16 ± 6.12	80.34 ± 8.31
	MD	94.33 ± 3.57	92.33 ± 5.03	90.72 ± 7.72	69.26 ± 14.68
BDST	Control	94.76 ± 2.01	92.76 ± 2.40	88.12 ± 4.85	77.02 ± 9.58
	MD	92.59 ± 5.09	90.52 ± 6.63	84.81 ± 12.25	70.97 ± 16.37
CBT	Control	95.64 ± 1.74	93.74 ± 2.93	92.10 ± 3.24	76.43 ± 8.82
	MD	94.00 ± 4.67	90.60 ± 7.82	88.59 ± 9.27	71.91 ± 19.03
MRT	Control	94.71 ± 2.33	93.36 ± 2.95	90.45 ± 4.88	77.79 ± 10.12
	MD	92.72 ± 5.33	90.24 ± 6.31	86.91 ± 10.81	71.52 ± 18.18
SDCT	Control	94.45 ± 1.86	92.74 ± 2.62	89.53 ± 5.49	77.19 ± 10.49
	MD	94.34 ± 2.93	92.67 ± 3.31	88.07 ± 7.39	71.28 ± 14.53
SAT	Control	94.93 ± 1.75	92.84 ± 2.70	90.12 ± 4.95	77.72 ± 12.28
	MD	93.22 ± 4.06	90.28 ± 6.29	88.36 ± 7.01	71.76 ± 17.06

*Note.* X; Mean, VST = Visual Stroop Task; BDST = Backward Digit Span Task; CBT = Corsi Block Task; MRT = Mental Rotation Task; SDCT = Symbol Digit Coding Task; SAT = Shifting Attention Task. Values are presented as Mean ± SD. C1, C3, C4, and C6 refer to specific SOT conditions, characterized by increasing postural difficulty.

The LMM analysis revealed significant main effects of Condition [*F*(3, 1512) = 698.79, *p* < 0.001, $\eta_{p}^{2}$ = 0.58] and Education [*F*(1, 54) = 5.95, *p* = 0.018, $\eta_{p}^{2}$ = 0.10]. Age was not a significant predictor of balance performance (*p* = 0.88). A main effect of Group approached statistical significance [*F*(1, 54) = 3.76, *p* = 0.058, $\eta_{p}^{2}$ = 0.07]. A significant Group by Condition interaction was also found [*F*(1, 1512) = 13.64, *p* < 0.001, $\eta_{p}^{2}$ = 0.03]. Post-hoc analysis showed that while the balance scores of the two groups were not significantly different in the easier sensory conditions (C1, C3, and C4; all *p* > 0.05), the MD group’s balance performance was significantly worse than the control group’s in the most challenging condition, C6 (*p* < 0.001). No other main effects or interactions were significant, including the three-way interaction of Group, Task, and Condition. Results are detailed in Table 5.

**Table 5.** Summary of LMM for balance performance.

Effect	F-statistic	*p*-value	$\eta_{p}^{2}$
Main effects			
Group	*F*(1, 54) = 3.76	0.058	0.07
Task	*F*(6, 1512) = 1.57	0.153	<0.01
Condition	*F*(3, 1512) = 698.79	<0.001**	0.58
Education	*F*(1, 54) = 5.95	0.018*	0.10
Age	F(1, 54) = 0.02	0.878	<0.01
Interaction effects			
Group × Task	*F*(6, 1512) = 0.42	0.864	<0.01
Group × Condition	*F*(3, 1512) = 13.64	<0.001**	0.03
Task × Condition	*F*(18, 1512) = 0.54	0.939	<0.01
Group × Task ×	*F*(18, 1512) = 0.77	0.739	<0.01

*Note.* F-statistics are reported as F(numerator df, denominator df). The “Task” factor includes seven levels: one single-task condition and six dual-task conditions. **p < 0.01.

We conducted further analyses to investigate the potential influence of clinical factors within the MD group. No significant correlations were found between disease duration and composite balance scores (*rho* = 0.10, *p* = 0.62) or any of the cognitive performance measures. Furthermore, when comparing patients with unilateral (n = 16) versus bilateral (n = 13) MD, no significant differences were found in composite balance scores (*W* = 88.5, *p* = 0.51). Similarly, no significant differences were found between these subgroups in reaction times or accuracy rates across any of the cognitive tasks (all *p* > 0.12).

## Discussion

The present study investigated cognitive-motor interference in individuals with MD using a dual-task paradigm. Our primary findings reveal a high dual-task cost in the MD group. The observed cost manifested not as a simple performance decline, but as a failure to adapt. This was evident in the finding that while healthy controls improved their visuospatial processing speed (MRT) under increasing postural load, MD patients could not. For executive function (SAT), a more subtle but significant disruption in the performance pattern was observed. Critically, the resulting cognitive load culminated in a disproportionate collapse of postural control in MD patients, specifically when sensory conflict was highest. These results support our central hypothesis that the compromised vestibular system in MD imposes a substantial cognitive load, which becomes evident as a performance deficit when attentional resources must be shared between maintaining balance and performing a concurrent cognitive task.

### Cognitive-motor interference in MD

Our results align with the literature, summarized in a recent systematic review by Li et al. (2024), which confirms a robust link between vestibular disorders and impairments in visuospatial processing, attention, and executive function.^30^ They are also directly consistent with the findings of Danneels et al. (2023), who identified a high dual-task cost in patients with bilateral vestibulopathy (BVP), most notable in a mental rotation task.^3^ The vulnerability of visuospatial skills across various vestibular pathologies highlights the crucial role of the vestibulo-cortical network in spatial cognition.

While our study established the presence of cognitive deficits in MD, other research highlights the specificity of these impairments. A study by Ahmad et al. (2022) demonstrated this specificity by comparing performance on a visuospatial memory task (CBT) with that on an auditory memory task (digit span). They found that patients with vestibular loss were significantly impaired on the visuospatial task but performed identically to controls on the auditory task, suggesting the cognitive consequences of vestibular loss are not global but are highly specific to the visuospatial domain.^31^ Their finding provides a strong rationale for the significant interference effects we observed on the MRT.

However, the extent of cognitive impairment may be modulated by the severity of the vestibular deficit. Popp et al. (2017) revealed a graded effect, where patients with unilateral vestibular failure showed deficits primarily in visuospatial and processing speed domains, whereas patients with bilateral vestibular failure exhibited more widespread impairments across all tested domains, including executive function.^32^ Similarly, Grabherr et al. (2011) found that bilateral vestibular failure, but not compensated unilateral vestibular failure, significantly impaired performance on mental rotation tasks.^33^ Our analysis did not reveal a significant difference between unilateral and bilateral MD patients. This may reflect the unique, fluctuating pathophysiology of MD compared to stable vestibular loss, or it may underscore the substantial cognitive burden that even unilateral MD imposes under a demanding dual-task load. This might further suggest that the mere presence of a fluctuating, unreliable vestibular signal, regardless of its source from one or two labyrinths, is sufficient to overwhelm the brain’s adaptive resources. Further supporting the importance of symptom chronicity, Pyykkö et al. (2024) demonstrated that MD patients with constant dizziness report more severe cognitive and postural problems than those with purely episodic vertigo, which suggests that a state of continuous sensory conflict is a key driver of cognitive load.^34^

### The neural and physiological basis of cognitive load in MD

The cognitive burden we have quantified is substantiated by evidence of functional and structural brain changes in patients with vestibular disorders. Russell et al. (2003) demonstrated that rats with complete bilateral vestibular lesions exhibit severe, long-term deficits in hippocampal-dependent spatial learning, confirming that intact vestibular input is crucial for the neural architecture of spatial cognition.^35^ For MD patients, the link between vestibular input and spatial cognition is made concrete by the work of Jian et al. (2024), who demonstrated that late-stage MD is associated with significant hippocampal atrophy, the degree of which correlates with the severity of endolymphatic hydrops.^7^ Such progressive neurodegeneration provides a clear anatomical substrate for the visuospatial deficits we observed.

Functionally, this peripheral pathology has cascading effects on central networks. The core vestibular hub in the parietal operculum shows abolished or reduced activity in response to vestibular stimulation in patients, as demonstrated by Ibitoye et al. (2023).^36^ The failure of a primary integrator likely forces the brain to compensate by recruiting more general-purpose cognitive networks. Indeed, EEG data from Li et al. (2024) show that MD patients exhibit increased activity in the saliency network, reflecting a state of active, effortful cognitive compensation.^37^ This compensatory state is metabolically costly, as supported by physiological data from Francavilla et al. (2024), who used pupillometry to demonstrate that MD patients exhibit a significantly larger pupillary response during simple postural tasks.^38^

The downstream consequence of hub failure and compensatory effect is a widespread disorganization of large-scale brain networks. A resting-state fMRI study by Li et al. (2023) found significantly decreased connectivity within the ventral attention network in patients with MD.^39^ This finding provides a direct neural correlation for the performance deficits we observed on the executive-based SAT. Taken together, the evidence suggests a system where peripheral damage leads to central structural and functional reorganization, resulting in a baseline state of high cognitive effort and reduced network efficiency, which in turn creates a processing bottleneck under dual-task load.

### The contrast with healthy systems and methodological considerations

The pathological nature of cognitive-motor interference in our MD cohort is brought into sharp relief when compared to the performance of healthy individuals. In healthy systems, a concurrent cognitive task can improve postural stability.^40–42^ Such improvement is due to a physical stiffening strategy, as well as a shift in attentional focus that enables more efficient, automatic postural control to emerge.^41,43^ The stabilizing effect is most pronounced when the postural task is sufficiently complex, which suggests it is a strategy the healthy brain deploys when needed.^42,43^ Moreover, the improvement in reaction time likely reflects an increase in general physiological arousal or alertness triggered by the postural challenge, which speeds up processing. In some cases, the dual-task context can even lead to mutual facilitation, where performance on both the cognitive and balance tasks improves.^44^ The neurophysiological basis for this appears to be an active upregulation of vestibular gain, as shown by McGeehan et al. (2017). The healthy brain’s strategy for successful dual-tasking is to rely more heavily on the vestibular system to automate posture, thereby freeing up cognitive resources.^45^ Our findings provide direct behavioral evidence for this phenomenon. Specifically, our healthy group significantly improved their reaction time on the demanding MRT precisely as postural challenges increased.

In contrast, MD patients are fundamentally unable to employ this efficient compensatory strategy because their vestibular system is unreliable. Unable to upregulate a faulty pathway, they incur a performance cost that healthy individuals might not experience. Our experimental design was tailored to expose such costs. By utilizing CDP, we employed a tool that provides a sensitive, quantitative, and functional measure of the ability to resolve sensory conflict.^46^ Furthermore, as a study by Beacham (2021) demonstrated, dual-task outcomes are critically dependent on instructions. We explicitly instructed participants to prioritize posture, creating a standardized posture-first attentional policy.^47^ This ensures that the observed decrements in cognitive performance are a direct reflection of the resources consumed by the postural task. Neither group showed an apparent reduction in postural stability under dual-task settings when compared to the single-task baseline, which is consistent with the posture-first strategy. The maintenance of stability, however, came at a cost. For the MD group, preserving balance required a reallocation of attentional resources that disproportionately slowed down performance on concurrent cognitive tasks.

In addition, age and education emerged as significant predictors of performance on several cognitive tasks and balance measures. This aligns with the concept of cognitive reserve, whereby higher education may buffer against cognitive demands in dual-task scenarios, while age exacerbates vestibular-related cognitive declines. For example, Smith et al. (2024) indicate that in vestibular disorders, age-related vestibular loss contributes to cognitive impairment, potentially mitigated by factors like education and environmental enrichment that enhance cognitive reserve through brain plasticity and reduced social deprivation.^48^ It is important to note that while age and education were significant covariates, their inclusion in the models did not negate our primary findings regarding dual-task interference. The finding confirms that observed deficits in the MD group are attributable to the vestibular pathology itself, rather than demographic disparities.

The sensitivity of the assessment paradigm is crucial. A study by Demirhan and Celebisoy (2023) found no cognitive deficits in a select group of MD patients who had no measurable vestibular loss between attacks, as assessed by single-task neurophysiological measures.^49^ Their finding suggests that standard single-task tests may not be sensitive enough to unmask the latent cognitive effort and that the deficits only become behaviorally evident when the system is stressed, as in our dual-task paradigm.

### Clinical implications

The differentiation between latent effort and overt performance deficits has significant clinical implications. Dornhoffer et al. (2021) found that while MD patients’ subjective dizziness scores improved with treatment, their self-reported cognitive complaints (CFQ scores) did not, which suggests a critical dissociation between vestibular symptoms and the cognitive burden of the disease.^50^ This highlights the need for objective, performance-based measures, such as the dual-task paradigm used in our study, to capture the functional impairment in MD fully.

Encouragingly, the functional impairment may be treatable. A longitudinal study by Zhong et al. (2023) found that effective treatment for vertigo resulted in significant improvements in objective cognitive scores, and these improvements were correlated with a reduction in vestibular handicap.^51^ Such a correlation positions vestibular dysfunction as a modifiable risk factor for cognitive decline. The population-level significance of the issue cannot be overstated. An epidemiological study by Semenov et al. (2016) found that vestibular loss was associated with a cognitive deficit equivalent to nearly 5 years of aging and was a stronger mediator of age-related cognitive decline than hearing or vision loss.^52^

### Limitations

The current study has some limitations. Cross-sectional design prevents inferences about causality or the long-term progression of cognitive-motor interference. Our sample size, while powered for our primary hypotheses, limits the statistical power of subgroup analyses, such as comparing unilateral versus bilateral disease. While we controlled for age and education, other factors, such as baseline fatigue or anxiety, could also influence performance. Lastly, although all patients with MD have some degree of hearing loss, its specific contribution to the cognitive load in our paradigm, while considered, cannot be fully disentangled from the vestibular component.

### Conclusion

The present study demonstrates a significant and quantifiable cognitive-motor interference cost in patients with MD, which is particularly evident in tasks that require visuospatial and executive functions. This deficit reflects a functional overload of a system already strained by the high cognitive demand of managing a compromised vestibular system. Our findings demonstrate that such a failure is a direct consequence of an inability to engage the efficient, adaptive postural strategies used by healthy individuals, resulting in a critical loss of stability only when sensory challenges are at their maximum. The cognitive burden of MD is a standalone, measurable, and clinically relevant feature of the disease that is linked to underlying neural changes but may be a modifiable risk factor. The objective assessment of cognitive-motor interactions is not only a critical component for current patient evaluation but also provides a therapeutic target for future rehabilitation strategies aimed at mitigating this functional overload and enhancing adaptive capacity in daily life.
